# Effects of sleep duration and changes in body mass index on diabetic kidney disease: a prospective cohort study

**DOI:** 10.3389/fendo.2023.1278665

**Published:** 2023-10-26

**Authors:** Cong Liu, Jia Zhang, Xing Wei, Juan Shi, Qianhua Fang, Weiwei Zhou, Lin Sun, Zhuomeng Hu, Jie Hong, Weiqiong Gu, Weiqing Wang, Ying Peng, Yifei Zhang

**Affiliations:** ^1^ Department of Endocrine and Metabolic Diseases, Shanghai Institute of Endocrine and Metabolic Diseases, Ruijin Hospital, Shanghai Jiao Tong University School of Medicine, Shanghai, China; ^2^ Shanghai National Clinical Research Center for Metabolic Diseases, Key Laboratory for Endocrine and Metabolic Diseases of the National Health Commission of the PR China, Shanghai Key Laboratory for Endocrine Tumor, State Key Laboratory of Medical Genomics, Ruijin Hospital, Shanghai Jiao Tong University School of Medicine, Shanghai, China

**Keywords:** sleep duration, obesity, changes in BMI, type 2 diabetes, diabetic kidney disease

## Abstract

**Aims:**

To examine the associations of sleep duration and changes in BMI with the onset of diabetic kidney disease (DKD).

**Materials and methods:**

2,959 participants with type 2 diabetes were divided into three groups based on sleep duration: short (<7 h/day), intermediate (7-9 h/day), or long (>9 h/day). Changes in BMI during follow-up were trisected into loss, stable, or gain groups. DKD was defined as either the urinary albumin/creatinine ratio (UACR) ≥ 3.39 mg/mmol or the estimated glomerular filtration rate (eGFR) < 60 mL/min/1.73m², or both. Cox regression models were used to assess hazard ratios (HRs) and 95% confidence intervals (CIs).

**Results:**

During a mean follow-up of 2.3 years, DKD occurred in 613 participants (20.7%). A J-shaped curve was observed between sleep duration and DKD. Compared to intermediate sleep duration, long sleep duration was associated with higher risks of DKD (HR 1.47; 95% CI: 1.19-1.81). In the joint analyses, compared to participants with intermediate sleep duration and stable BMI, long sleep duration with BMI gain had the highest risks of DKD (HR 2.04; 95% CI: 1.48-2.83). In contrast, short or intermediate sleep duration accompanied by decrease in BMI was associated with a reduced risk of DKD, with HRs of 0.50 (95% CI: 0.31-0.82) and 0.61 (95% CI:0.47-0.80), respectively.

**Conclusions:**

Long sleep duration is significantly associated with an increased risk of DKD, which is further amplified by obesity or BMI gain. These findings suggest that both proper sleep duration and weight control are essential to preventing DKD.

## Introduction

Diabetes affects 537 million adults globally in 2021 and is responsible for an estimated 2 million deaths, including those related to diabetic kidney disease (DKD) (1). It can lead to end-stage renal disease (ESRD) as kidney function deteriorates over time, ultimately necessitating interventions such as dialysis or kidney transplantation (2). The progression of DKD is heavily influenced by hyperglycemia, and common comorbidities such as hypertension and hyperlipidemia also play significant roles in the pathogenesis of DKD (3). These factors lead to glomerular endothelial dysfunction and podocyte damage, ultimately resulting in glomerulosclerosis and the formation of renal unit loss (4, 5). While the complex pathophysiological mechanisms involved are still being explored.

Sleep duration has been demonstrated as a critical factor influencing a variety of health outcomes like hyperglycemia, cardiovascular disease (CVD), and cognitive decline (6–8). The guidelines provided by the American Diabetes Association (ADA) indicate a correlation between inadequate or excessive sleep duration and elevated levels of glycated hemoglobin (HbA1c) (9). A previous cross-sectional study found both short and long sleep durations were associated with decreased estimated glomerular filtration rate (eGFR) as well as increased albuminuria (10). Another cross-sectional study suggested that longer daytime sleep among individuals with type 2 diabetes was linked to albuminuria (11). However, these cross-sectional studies cannot establish a causal relationship between sleep duration and DKD. Moreover, there is a notable scarcity of prospective cohort studies that have specifically investigated this association. Additionally, prior investigations have demonstrated a positive correlation between higher BMI and DKD, whereas a reduction in BMI has been linked to a decreased risk of DKD (12). This indicates that alterations in BMI contribute significantly to the onset of DKD. There is a growing recognition of the significance of comprehensive management of lifestyle and metabolic health status. Both of these aspects are incorporated as modifiable factors in the care goals for individuals with diabetes. It is highly meaningful to perform risk stratification for DKD by combining sleep duration and changes in BMI as it enhances the identification of high-risk populations and enables targeted interventions.

Therefore, the present study aimed to extend the existing knowledge by investigating the relationship between sleep duration and DKD, and exploring the potential influence of obesity and changes in BMI on this association.

## Materials and methods

### Study design and participants

This prospective cohort was part of a pilot and standard system, the National Metabolic Management Center (MMC) at Ruijin Hospital, Shanghai Jiao Tong University School of Medicine. The details of MMC have been previously described (13, 14). A total of 5,655 individuals aged 18 years or older, who had received a confirmed diagnosis of type 2 diabetes, were followed from June 2017 to December 2022. Out of these individuals, we excluded participants with a follow-up period of fewer than 6 months (n = 750), existing DKD at baseline (n = 1,327), or lacking information on sleep duration (n = 318), urinary albumin/creatinine ratio (UACR), or eGFR (n = 301). The final analysis comprised a total of 2,959 participants (the flowchart of participant inclusion appears in **Supplementary Figure 1**). Written informed consent was obtained from each participant, and the study protocol received ethical approval from the Institutional Review Board of Ruijin Hospital, Shanghai Jiao Tong University School of Medicine.

### General characteristics and outcomes

Participants underwent an extensive physical examination, blood sample collection, and completion of baseline questionnaires to obtain lifestyle-related information. The questionnaires included questions on night sleep duration, nap duration, and sleep quality. Participants’ total sleep duration of twenty-four hours was obtained by combining both nocturnal and midday sleep periods. Three self-reported options were available to assess sleep quality based on subjective evaluation: 1) good, 2) poor, and 3) requiring medication. The definition of ideal smoking status and alcohol consumption, as well as the standards for physical measurements, were detailed in our previously published studies (15, 16). Blood pressure was measured after a minimum of 5 minutes of rest. Blood samples were collected from each participant following an overnight fasting period to obtain HbA1c, fasting plasma glucose (FPG) and 2-hour post-load plasma glucose (PG), lipid profile, and other laboratory parameters. The UACR was determined by dividing the concentration of urinary albumin by the concentration of urinary creatinine. The eGFR was calculated using the Chronic Kidney Disease Epidemiology Collaboration (CKD-EPI) equation to assess renal function (17).

### Definitions

Based on previous studies, the classification of sleep duration was as follows: intermediate (7-9 h/day), short (<7 h/day), and long (>9 h/day) (18). BMI was divided into three categories according to the criteria recommended by the National Health Commission of the People’s Republic of China: normal weight, <24 kg/m^2^; overweight, 24-27.9 kg/m^2^ and obese, ≥28 kg/m^2^. Central obesity was defined as waist circumference ≥90 cm for men and ≥85 cm for women (19). To perform the joint analysis, we categorized the study participants based on their changes in BMI from baseline to the final visit. Participants with the lowest one-third change in BMI were classified as the “loss” group; those with the middle one-third change were classified as the “stable” group; and the upper thirds were classified as the “gain” group (20). Considering the potential impact of baseline BMI status on DKD, we categorized participants into four groups based on changes in BMI status: “Remained normal BMI” for those with normal BMI at both baseline and the last examination, “Remained overweight or obese” for those who were overweight or obese at both examinations, “Became normal BMI” for those who were overweight or obese at baseline but had a normal BMI at the last examination, and “Became overweight or obese” for those with a normal BMI at baseline but were overweight or obese at the last examination. The details of the characteristics of BMI changes in the different groups, categorized based on changes in BMI or BMI status, have been provided in the Appendix (**Table S1**). The definition of diabetes was fasting plasma glucose ≥7.0 mmol/L, or 2-hour plasma glucose ≥11.1 mmol/L, or HbA1c ≥6.5%, or previously diagnosed by their healthcare provider (21). Albuminuria was defined as UACR ≥3.39 mg/mmol. The presence of albuminuria or eGFR <60 mL/min/1.73m^2^ was defined as the presence of DKD (22).

### Statistical analyses

Continuous variables were presented as mean ± standard deviation or median with interquartile range, while categorical variables were presented as counts and proportions. To assess differences among groups, P-values were calculated using the ANOVA method for continuous variables and chi-square tests for categorical variables.

We employed Cox proportional hazards models to investigate the associations between sleep duration and the incidence of DKD. The Schoenfeld residuals method was utilized to evaluate the proportional hazards assumptions of the Cox models. According to previous studies on risk factors for kidney diseases, we selected potential confounding factors and adjusted for them in the multivariable model (23–25). Model 1 adjusted for age and sex, while in Model 2, additional adjustments were made for the duration of diabetes, HbA1c, smoking status, alcohol intake, BMI, eGFR, use of angiotensin-converting enzyme inhibitor (ACEI) or angiotensin receptor blockers (ARB); model 3 was further adjusted for history of hypertension, CVD and cancer, and sleep quality (good or poor/requiring medication) based on model 2. Restricted cubic splines with three knots placed at the 5th, 50th, and 95th percentiles were employed to examine the associations between sleep duration and DKD. We performed stratified analyses by age (<50 years, 50-60 years, or >60 years), sex, duration of diabetes (<5 years, or ≥5 years), glycemic control (HbA1c <7%, or ≥7%), BMI (<24 kg/m^2^, 24-27.9 kg/m^2^, or ≥28 kg/m^2^), changes in BMI (stable, loss, or gain), changes in BMI status (remained normal, remained overweight or obese, became normal, or became overweight or obese), and waist circumference (normal, or central obesity). Adjustments to the model were the same as in model 3 except for the stratification variables. The statistical significance of interactions was assessed using the likelihood ratio test, comparing models with and without cross-product terms between sleep duration categories (<7, 7-9, >9 h/day) and the stratification variables. To examine whether the association between sleep duration and DKD was modified by BMI, changes in BMI, or changes in BMI status, we conducted joint analyses. The model accounted for the covariates included in model 3, except for BMI.

Sensitivity analyses were conducted. Initially, the participants were categorized into three groups according to the percentage variation in BMI to test the robustness of the results: Individuals with a BMI reduction of 5.0% or higher were designated as the “BMI loss” group (n=636). This threshold aligns with the weight loss recommendation of ≥5% for individuals who are overweight or obese and diagnosed with type 2 diabetes, as routinely advocated by the American Diabetes Association (ADA) (26). As BMI decreased by an average of 1.3% for all participants during the follow-up period while the number of participants with a ≥ 5% increase in BMI was less than one in ten (n=286), the “BMI gain” group consisted of individuals who experienced a BMI increase of 3.0% or higher (n = 495). The participants in the “BMI stable” group had a BMI decrease of < 5.0% or an increase of < 3.0% (n=1805). Furthermore, we analyzed the effect of nighttime sleep on DKD with an additional adjustment for the duration of daytime naps.

The statistical analysis was conducted using R version 4.1.1 (R Foundation, Vienna, Austria). Two-tailed P-values were reported, and a significance level of 0.05 was used to determine statistical significance.

## Results

### Baseline characteristics

The baseline characteristics of the study participants according to sleep duration are presented in **Table 1**. The mean age of the participants was 55.2 (SD, 11.7) years, and 1,765 were males (59.7%). Of all the 2,959 participants involved, 15%, 70%, and 15% reported sleeping <7 hours, 7-9 hours, and >9 hours per day, respectively. Participants who reported sleeping >9 hours per day were slightly older with a longer duration of diabetes and poorer glycemic control compared to the other two groups (p < 0.05).

**Table 1:** Baseline characteristics of the study population according to sleep duration.

		Habitual sleep duration, h/day			
	Total	< 7	7 - 9	> 9	P
	(N=2,959)	(N=443)	(N=2,073)	(N=443)	
Age, years	55.19 ± 11.74	54.47 ± 11.17	55.02 ± 11.80	56.73 ± 11.90	0.008
Males	1765 (59.65%)	247 (55.76%)	1248 (60.20%)	270 (60.95%)	0.186
Duration of diabetes, years	7.49 ± 7.41	7.39 ± 7.36	7.28 ± 7.34	8.58 ± 7.69	0.003
Education, high school and above, n (%)	2199 (74.80%)	328 (74.55%)	1545 (75.11%)	326 (73.59%)	0.793
History of hypertension, n (%)	1148 (38.93%)	154 (34.92%)	809 (39.16%)	185 (41.86%)	0.099
History of cardiovascular disease, n (%)	359 (12.19%)	52 (11.82%)	233 (11.29%)	74 (16.74%)	0.006
History of any cancer, n (%)	135 (4.58%)	19 (4.32%)	93 (4.51%)	23 (5.20%)	0.784
Ideal smoking status, n (%)	2278 (77.22%)	346 (78.28%)	1597 (77.26%)	335 (75.96%)	0.712
Drinking, n (%)	279 (9.44%)	46 (10.38%)	195 (9.42%)	38 (8.60%)	0.661
Systolic blood pressure, mmHg	126.38 ± 16.56	126.12 ± 17.24	126.48 ± 16.52	126.15 ± 16.07	0.870
Diastolic blood pressure, mmHg	73.56 ± 10.42	73.94 ± 10.95	73.65 ± 10.35	72.74 ± 10.19	0.177
BMI, kg/m^2^	25.60 ± 3.99	25.93 ± 4.63	25.57 ± 3.86	25.38 ± 3.90	0.104
Waist circumference, cm	91.19 ± 10.17	91.63 ± 11.15	91.10 ± 9.93	91.17 ± 10.25	0.618
HbA1c, %	7.71 ± 1.68	7.54 ± 1.59	7.71 ± 1.69	7.89 ± 1.67	0.009
HbA1c < 7%, n (%)	1190 (40.37%)	189 (42.76%)	851 (41.23%)	150 (33.94%)	0.010
Fasting blood glucose, mmol/L	8.83 ± 3.12	8.73 ± 2.82	8.83 ± 3.18	8.94 ± 3.11	0.605
2-h postload glucose, mmol/L	14.81 ± 4.80	14.62 ± 4.68	14.72 ± 4.77	15.40 ± 5.00	0.019
Triglycerides, mmol/L	1.47 [1.03;2.11]	1.51 [1.04;2.23]	1.45 [1.02;2.11]	1.48 [1.07;2.03]	0.304
Total cholesterol, mmol/L	4.95 ± 1.18	4.97 ± 1.10	4.96 ± 1.17	4.90 ± 1.28	0.635
HDL cholesterol, mmol/L	1.26 ± 0.32	1.25 ± 0.31	1.26 ± 0.33	1.24 ± 0.31	0.400
LDL cholesterol, mmol/L	3.05 ± 0.97	3.07 ± 0.94	3.05 ± 0.96	3.03 ± 1.05	0.863
ACEI/ARB, n (%)	791 (27.75%)	103 (24.52%)	553 (27.57%)	135 (31.84%)	0.056

Data are shown as mean ± SD or median (interquartile range) for continuous variables and count (percentage) for categorical variables. P values refer to comparisons among groups using the ANOVA statistical method for comparing continuous variables and the chi-square tests for comparing categorical variables.

BMI, body mass index; HbA1c, glycated hemoglobin; HDL, high-density lipoprotein; LDL, low-density lipoprotein;

ACEI, angiotensin-converting enzyme inhibitor; ARB, angiotensin receptor blockers.

### Independent association of sleep duration with DKD and albuminuria

Throughout a mean ( ± SD) follow-up period of 2.3 (± 1.4) years, a total of 613 incidents (20.7%) of DKD were detected. **Table 2** demonstrates a significant association between sleep duration and the risks of DKD. Participants with long sleep duration had higher risks of DKD (26.4%) compared to those with intermediate (19.5%) and short sleep duration (20.5%) (P = 0.006). Similarly, the incidence of albuminuria was higher in participants with long sleep duration, with 23.5% developing albuminuria compared to 16.6% in the reference group and 19.2% in the short sleep duration group (P = 0.003).

**Table 2:** Associations of sleep duration with DKD among people with type 2 diabetes.

	Habitual sleep duration, h/day			
	< 7	7 - 9	> 9	P
**DKD incidence, n (%)**	91 (20.54%)	405 (19.54%)	117 (26.41%)	0.006
Model 1	1.01 (0.80-1.26)	1.00 (Ref)	1.45 (1.18-1.79)	
Model 2	0.96 (0.75-1.23)	1.00 (Ref)	1.47 (1.19-1.82)	
Model 3	0.97 (0.75-1.24)	1.00 (Ref)	1.47 (1.19-1.81)	
**Albuminuria incidence, n (%)**	85 (19.19%)	345 (16.64%)	104 (23.48%)	0.003
Model 1	1.10 (0.87-1.40)	1.00 (Ref)	1.54 (1.24-1.92)	
Model 2	1.04 (0.81-1.34)	1.00 (Ref)	1.59 (1.27-1.99)	
Model 3	1.06 (0.81-1.37)	1.00 (Ref)	1.55 (1.24-1.95)	

Hazard ratios and 95% confidence intervals of the associations of sleep duration with DKD and albuminuria. Model 1 was adjusted for age and sex; model 2 was further adjusted for duration of diabetes, HbA1c, smoking status, alcohol intake, BMI, eGFR, use of angiotensin-converting enzyme inhibitor (ACEI) or angiotensin receptor blockers (ARB) based on model 1; model 3 was further adjusted for history of hypertension, CVD and cancer, and sleep quality (good or poor/requiring medication) based on model 2.

**Figure 1:**
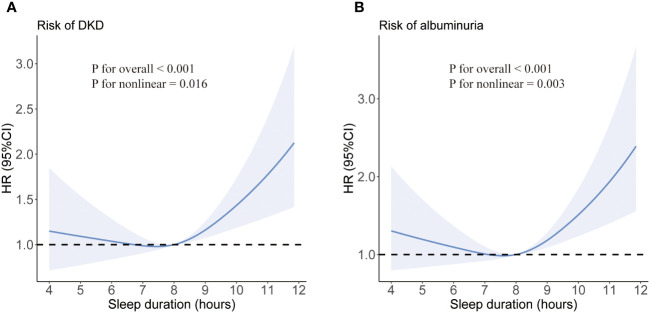
Multivariate-adjusted spline curves for associations of sleep duration with DKD **(A)** and albuminuria **(B)**. Sleep duration was fitted as a smooth term using a restricted cubic spline with 3 knots. Shading indicates 95% confidence intervals. The model was adjusted for age, sex, duration of diabetes, HbA1c, smoking status, alcohol intake, BMI, eGFR, use of ACEI/ARB, history of hypertension, CVD and cancer, and sleep quality.

We did not detect any evidence of a violation of the proportional hazard assumption, employing a p-value threshold of 0.05 (**Figure S2**). After adjusting for age and gender, long sleep duration was associated with an increased risk of developing DKD (HR 1.45; 95% CI:1.18-1.79) compared with intermediate sleep duration (model 1). The association was prominent after additional adjustment for diabetes duration, smoking status, alcohol intake, BMI, eGFR, and use of ACEI/ARB (HR 1.47; 95% CI:1.19-1.82) (model 2). Furthermore, the observed association between longer sleep and risk of DKD remained significant after controlling for sleep quality and history of hypertension, CVD, and cancer (HR 1.47; 95% CI: 1.19-1.81) (model 3). Short sleep duration did not show an association with increased risks of DKD or albuminuria. The restricted cubic spline regression analysis confirmed a J-shaped curve, with >9 h/day of sleep being associated with a higher risk of DKD and albuminuria (**Figure 1**). The same covariates as in Model 3 were included in the analysis. In addition, consistent results were observed in subgroup analyses (all interaction P-values > 0.05) (see **Figure S3**).

### Joint association of sleep duration and BMI with DKD and albuminuria

**Figure 2:**
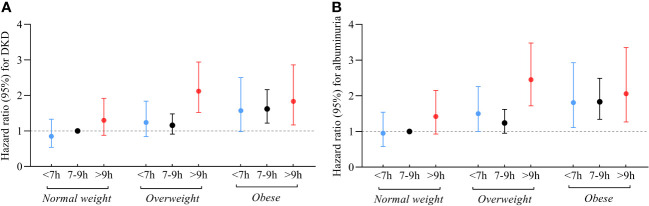
Relationship between sleep duration and risk of DKD **(A)** and albuminuria **(B)** among participants with varying BMI. Those who slept for 7-9 hours/day and had a BMI < 24 kg/m^2^ were referenced. All models were adjusted for age, sex, duration of diabetes, HbA1c, smoking status, alcohol intake, eGFR, use of ACEI/ARB, history of hypertension, CVD and cancer, and sleep quality.

**Figures 2A, B**; **Table S2** show the joint association of sleep duration and BMI with DKD and albuminuria. Individuals with long sleep duration and overweight had higher risks of DKD (HR 2.12; 95% CI: 1.52-2.94) and albuminuria (HR 2.45; 95% CI: 1.72-3.48) compared to the reference group, which consists of individuals with intermediate sleep duration and BMI < 24kg/m^2^. Similarly, individuals with long sleep duration and obesity were at higher risk of DKD (HR 1.83; 95% CI: 1.17-2.86) and albuminuria (HR 2.06; 95% CI: 1.27-3.35) compared to the reference group. Additionally, participants with short or intermediate sleep duration and obesity were also associated with an elevated risk of DKD and albuminuria.

### Joint association of sleep duration and changes in BMI with DKD and albuminuria

**Figure 3:**
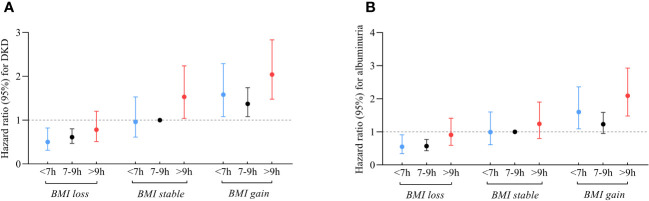
Relationship between sleep duration and risk of DKD **(A)** and albuminuria **(B)** among participants with varying changes in BMI. Those who slept 7-9 hours/day and had stable BMI during follow-up were referenced. All models were adjusted for age, sex, duration of diabetes, HbA1c, smoking status, alcohol intake, eGFR, use of ACEI/ARB, history of hypertension, CVD and cancer, and sleep quality.

**Figures 3A, B**; **Table S3** illustrate the joint association of sleep duration and changes in BMI with DKD and albuminuria. Participants who had long sleep duration and experienced BMI gain faced the highest risks of DKD (HR 2.04; 95% CI: 1.48-2.83) and albuminuria (HR 2.09; 95% CI: 1.48-2.93) compared to the reference group, comprising individuals with intermediate sleep duration and stable BMI. In contrast, participants with short sleep patterns and experiencing BMI loss were found to have lower risks of DKD (HR 0.50; 95% CI: 0.31-0.82) and albuminuria (HR 0.55; 95% CI: 0.34-0.91) compared to the reference group. Similarly, participants with intermediate sleep patterns and BMI loss also had a protective effect on the development of DKD (HR 0.61; 95% CI: 0.47-0.80) and albuminuria (HR 0.57; 95% CI: 0.43-0.77).

### Joint association of sleep duration and changes in BMI status with DKD and albuminuria

**Figure 4:**
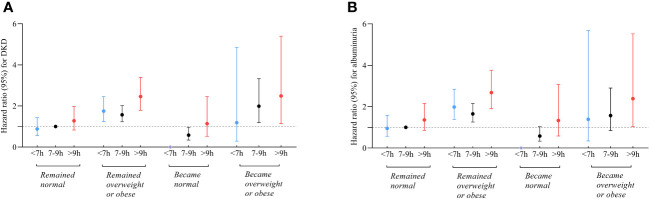
Relationship between sleep duration and risk of DKD **(A)** and albuminuria **(B)** among participants with varying changes in BMI status. Those who slept 7-9 hours/day and remained normal BMI during follow-up were referenced. All models were adjusted for age, sex, duration of diabetes, HbA1c, smoking status, alcohol intake, eGFR, use of ACEI/ARB, history of hypertension, CVD and cancer, and sleep quality. ^*^ The group with short sleep patterns that transitioned to a normal BMI had no participants who developed DKD.

**Figures 4A, B**; **Table S4** depict the joint association of sleep duration and changes in BMI status with DKD and albuminuria. Participants who had long sleep duration and became overweight or obese faced the highest risks of DKD (HR 2.49; 95% CI:1.14-5.40), in contrast to the reference group consisting of individuals with intermediate sleep durations and remained BMI normal. Similarly, participants who had long sleep durations and remained overweight or obese were also at an increased risk of DKD (HR 2.46; 95% CI: 1.79-3.39). Conversely, participants with intermediate sleep patterns who transitioned to a normal BMI exhibited reduced risks of DKD compared to the reference group (HR 0.58; 95%CI: 0.34-0.97). Furthermore, the group with short sleep patterns that transitioned to a normal BMI had no participants who developed DKD.

### Sensitivity analyses

The results remained largely consistent in all sensitivity analyses (**Figures S4-5**; **Table S5**).

## Discussion

This prospective cohort study examined the relationship between sleep duration and the occurrence of DKD in individuals diagnosed with T2DM. Our findings revealed a significant link between long sleep duration (>9 h/day) and an elevated risk of DKD, which remained after adjusting for potential confounding variables. We identified a nonlinear relationship between sleep duration and the onset of DKD, characterized by a J-shaped curve, which was also present in the occurrence of albuminuria. Furthermore, we found that participants with both long sleep duration and BMI gain faced the greatest risks of DKD compared to individuals with intermediate sleep duration and stable BMI. To our knowledge, this is the first prospective cohort to report that long sleep duration was associated with a higher risk of DKD when compared to those who slept 7-9 hours per day. Moreover, our results emphasize the influence of BMI changes on this association, suggesting that the combination of longer sleep and BMI gain may synergistically contribute to the development of DKD.

Although the association between long sleep duration and the risk of DKD has been proposed, relevant studies published thus far are sparse (10, 11). Several studies have examined the connection between sleep duration and CKD in the general population; however, the findings have yielded inconsistent results. A previous investigation identified a U-shaped relationship between both insufficient sleep duration (≤4 hours) and excessive sleep duration (>10 hours) and CKD in middle-aged and older people (27), while the Nurses’ Health Study (NHS) did not observe a correlation between longer sleep (≥9 hours) and rapid decline in eGFR (decrease of more than 30%) (28). Furthermore, research conducted in Japan discovered that longer sleep duration (>8 hours) was an important predictor of end-stage kidney disease (ESKD) (29). The disparities between our findings and the conclusions of previous research may be attributed, in part, to variations in race, geographical locations, underlying disease, and baseline renal function among these study populations.

The mechanisms involved in the negative effects of long sleep duration on the onset of DKD have not been sufficiently appreciated. To begin with, prolonged periods of sleep may trigger an upsurge in high-sensitivity C-reactive protein (hs-CRP) levels and stimulate an inflammatory response, which further leads to cellular injury, glomerular endothelial dysfunction, proliferation of mesangial cells, and increased vascular permeability (30, 31). These factors can ultimately contribute to the development of albuminuria and DKD (32). Second, longer sleep duration is associated with several metabolic abnormalities, including hypertension, dyslipidemia, and insulin resistance (33, 34), all of which are closely associated with the onset of DKD (35). Furthermore, numerous studies have reported that individuals with long sleep durations often have other unhealthy daily behaviors, such as sedentary behavior and lack of physical activity, which are well-established risk factors for diabetes complications, including DKD (36–38).

Consistent with previous studies suggesting that obesity is an independent risk factor for DKD (39), our study extends this understanding through joint analyses of sleep duration and variability in BMI and BMI status. In our study, the effect of long sleep duration on the incidence of DKD was more pronounced among those who experienced BMI gain, transitioned to overweight or obese, or remained overweight or obese. The biological mechanisms underlying the joint effects are still unclear. On one hand, previous studies have indicated that prolonged sleep has been associated with reduced physical activity, decreased energy expenditure, and subsequent weight gain (40). On the other hand, evidence suggests that obese individuals tend to have a higher risk of obstructive sleep apnea (OSA), a condition that adversely affects the quality of sleep and contributes to excessive daytime sleepiness (41, 42). This bidirectional association between sleep and obesity forms a detrimental cycle that exacerbates the risk of DKD. In addition, both long sleep duration and obesity are related to risk factors of DKD, like insulin resistance, hypertension, dyslipidemia, and inflammatory response (43, 44). On the contrary, participants with short or intermediate sleep duration and a decrease in BMI during follow-up were observed to be related to a reduction in the risk of DKD. The finding suggests that maintaining appropriate sleep duration and achieving weight loss may have a protective effect against DKD. A previous randomized controlled trial has demonstrated that weight loss ameliorates insulin resistance and results in lower HbA1c and systolic blood pressure in individuals with obesity, thereby delaying the onset of the microvascular complications of diabetes (45). This finding coincides with the outcomes of our analysis. Further exploration is needed to understand the underlying mechanisms of this relationship. Additionally, further research is warranted to establish specific and effective strategies targeting sleep duration and weight management to prevent or delay the onset and progression of DKD.

The strengths of our study include its large sample size and the implementation of a longitudinal study design, which provides a more robust approach compared to cross-sectional studies. Our finding extends previous research by exploring the combined effects of sleep duration and changes in BMI on the occurrence of DKD. Additionally, we fully adjusted for potential confounding factors to ensure the reliability of the outcomes. In particular, we adjusted for sleep quality to assess the independent risk of sleep duration for DKD, which has rarely been considered in previous studies.

Despite the novel insights provided by this study, several potential limitations should also be acknowledged. Firstly, although the association between sleep duration and BMI changes and DKD incidence reached statistical significance, our conclusions still need to be validated in populations of other genetic backgrounds. Secondly, our assessment of sleep duration is self-reported while those measured using polysomnography are more objective. However, self-reported questionnaires have been widely utilized as a more feasible form of epidemiological investigation in large-scale population studies.

## Conclusion

Long sleep duration was significantly associated with increased risks of DKD. Notably, compared to participants with intermediate sleep duration and stable BMI, long sleep duration with BMI gain had higher risks of DKD. Conversely, short or intermediate sleep duration with loss of BMI was associated with decreased risks of DKD, indicating that both appropriate sleep duration and weight control are required to prevent the development of DKD. Future investigations are warranted to elucidate the underlying mechanisms of this association and develop better intervention strategies for sleep habits and weight management.

## STROBE statement

This study was reported in accordance with STROBE guidelines for cohort studies.

## Data availability statement

The raw data supporting the conclusions of this article will be made available by the authors, without undue reservation.

## Ethics statement

The studies involving humans were approved by the Institutional Review Board of Ruijin Hospital, Shanghai Jiao Tong University School of Medicine. The studies were conducted in accordance with the local legislation and institutional requirements. The participants provided their written informed consent to participate in this study.

## Author contributions

CL: Conceptualization, Data curation, Visualization, Writing – original draft. JZ: Methodology, Validation, Writing – original draft. XW: Data curation, Methodology, Visualization, Writing – original draft. JS: Formal Analysis, Methodology, Writing – review & editing. QF: Conceptualization, Software, Writing – original draft. WZ: Data curation, Validation, Writing – review & editing. LS: Data curation, Investigation, Software. ZH: Data curation, Investigation, Validation. JH: Writing – review & editing. WG: Formal Analysis, Validation, Writing – review & editing. WW: Funding acquisition, Resources. YP: Supervision, Writing – review & editing. YZ: Funding acquisition, Supervision, Project administration.
