# Supplementary material for: Effects of sleep duration and changes in body mass index on diabetic kidney disease: a prospective cohort study
Source: Front Endocrinol (Lausanne). 2023 Oct 26;14:1278665. doi: 10.3389/fendo.2023.1278665 (PMC10641014; doi:10.3389/fendo.2023.1278665)
Supplement: Supplementary file 1 [file DataSheet_1.docx]

Supplementary Material

**Supplementary Figures and Tables**

[Figure S1. The flowchart of the study population 2](#_Toc146123185)

[Figure S2. The proportional hazard assumption 3](#_Toc146123186)

[Figure S3. Stratified analysis of the association between long sleep duration and DKD. 3](#_Toc146123187)

[Figure S4. Multivariate-adjusted spline curves for associations of nocturnal sleep duration with DKD (A) and albuminuria (B). 5](#_Toc146123188)

[Figure S5. Relationship between sleep duration and risk of DKD (A) and albuminuria (B) among participants with varying changes in BMI. 6](#_Toc146123189)

[Table S1. Characteristics of BMI changes in the different groups according to changes in BMI or BMI status 7](#_Toc146123190)

[Table S2. Risks of DKD and albuminuria according to sleep duration and BMI 8](#_Toc146123191)

[Table S3. Risks of DKD and albuminuria according to sleep duration and changes in BMI 9](#_Toc146123192)

[Table S4. Risks of DKD and albuminuria according to sleep duration and changes in BMI status 10](#_Toc146123193)

[Table S5. Risks of DKD and albuminuria according to sleep duration and changes in BMI 11](#_Toc146123194)

##
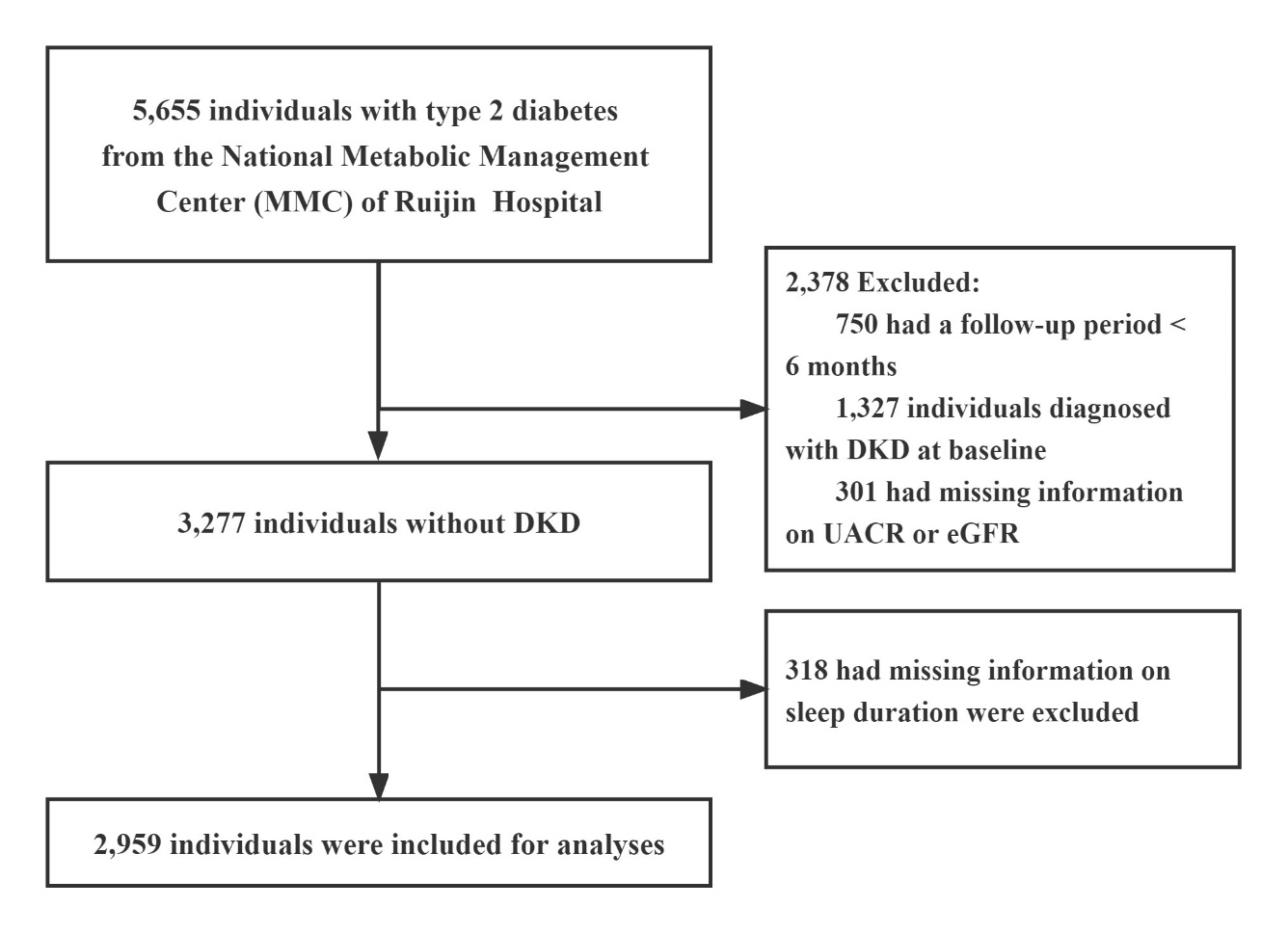
Figure S1. The flowchart of the study population

## Figure S2. The proportional hazard assumption

##
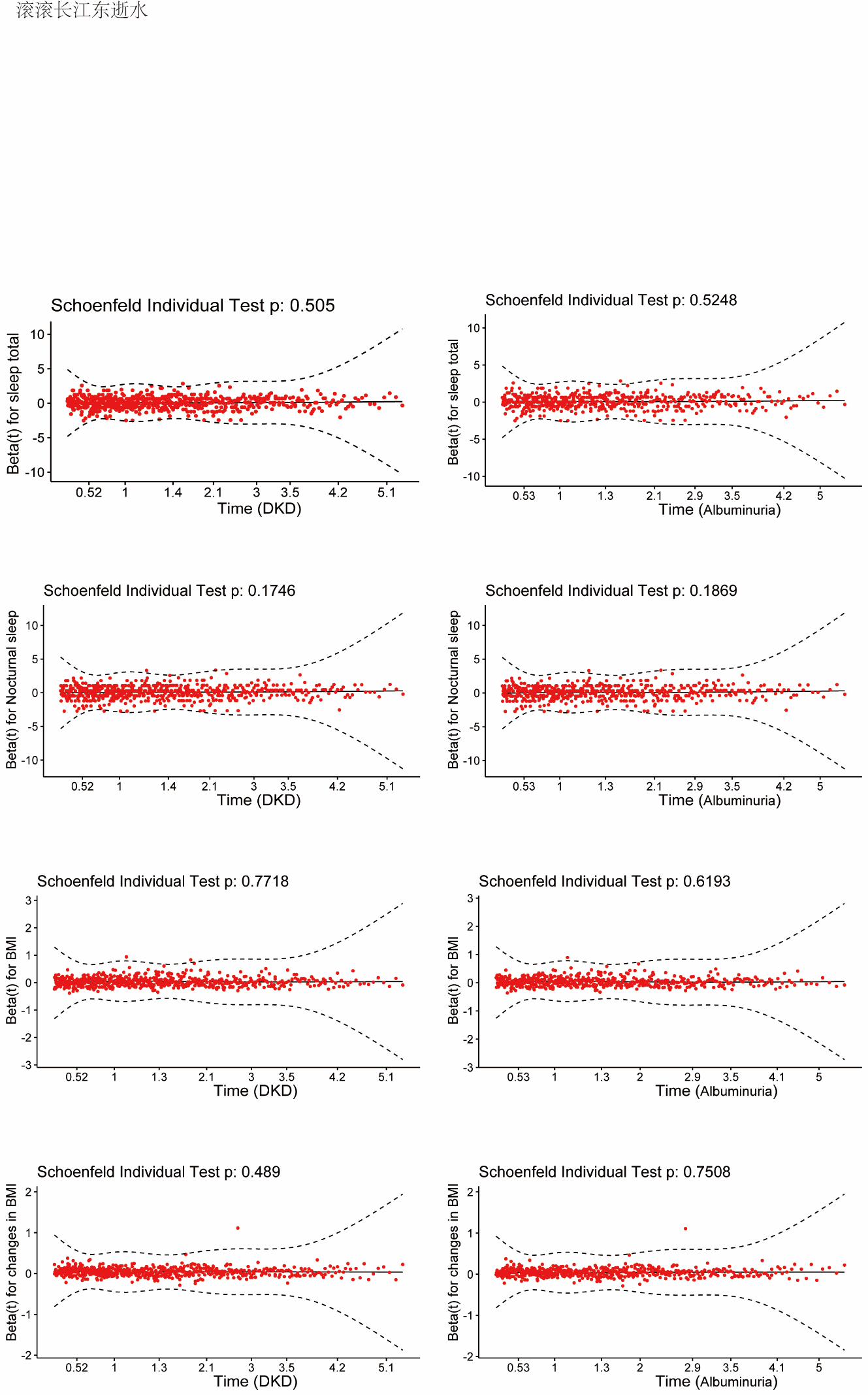
Figure S3. Stratified analysis of the association between long sleep duration and DKD.

^a^Overweight was defined as BMI between 24 and 27.9 kg/m^2^ and obese was defined as BMI ≥ 28 kg/m^2^.

^b^Central obesity was defined as waist circumference ≥ 90 cm in men and waist circumference ≥ 85 cm in women.

The forest plot displays the hazard ratios (HRs) and 95% confidence intervals (CIs) for individuals with > 9 hours of sleep, using 7-9 hours of sleep as the reference group. Models were adjusted for age, sex, duration of diabetes, HbA1c, BMI, smoking status, alcohol intake, eGFR, use of ACEI/ARB, and sleep quality. The stratification variable was not included in the model when stratifying by itself.

Abbreviations: HbA1c, glycated hemoglobin; BMI, body mass index


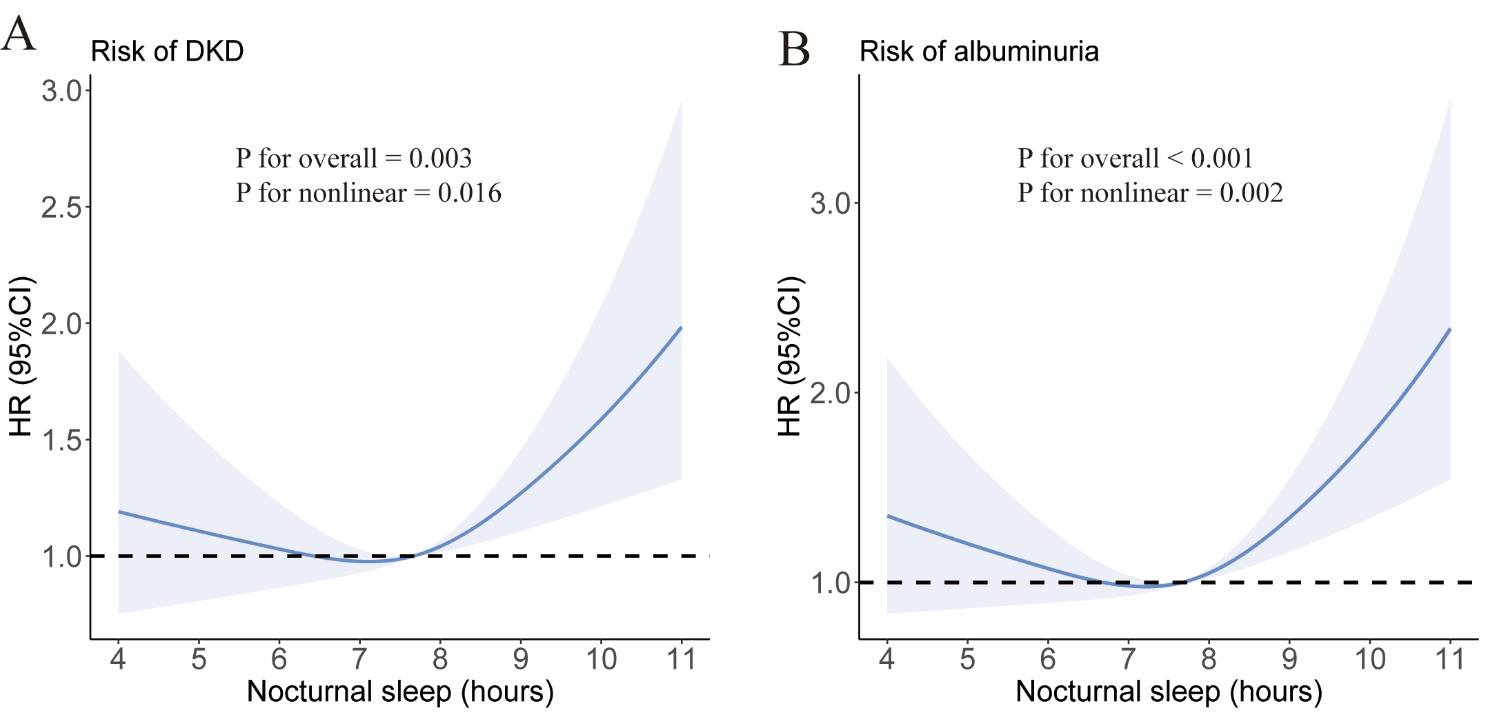
Figure S4. Multivariate-adjusted spline curves for associations of nocturnal sleep duration with DKD (A) and albuminuria (B).

Nocturnal sleep duration was fitted as a smooth term using a restricted cubic spline with 3 knots. Shading indicates 95% confidence intervals. The model was adjusted for age, sex, duration of diabetes, HbA1c, smoking status, alcohol intake, BMI, eGFR, use of ACEI/ARB, history of hypertension, CVD and cancer, sleep quality and the duration of daytime naps.

**
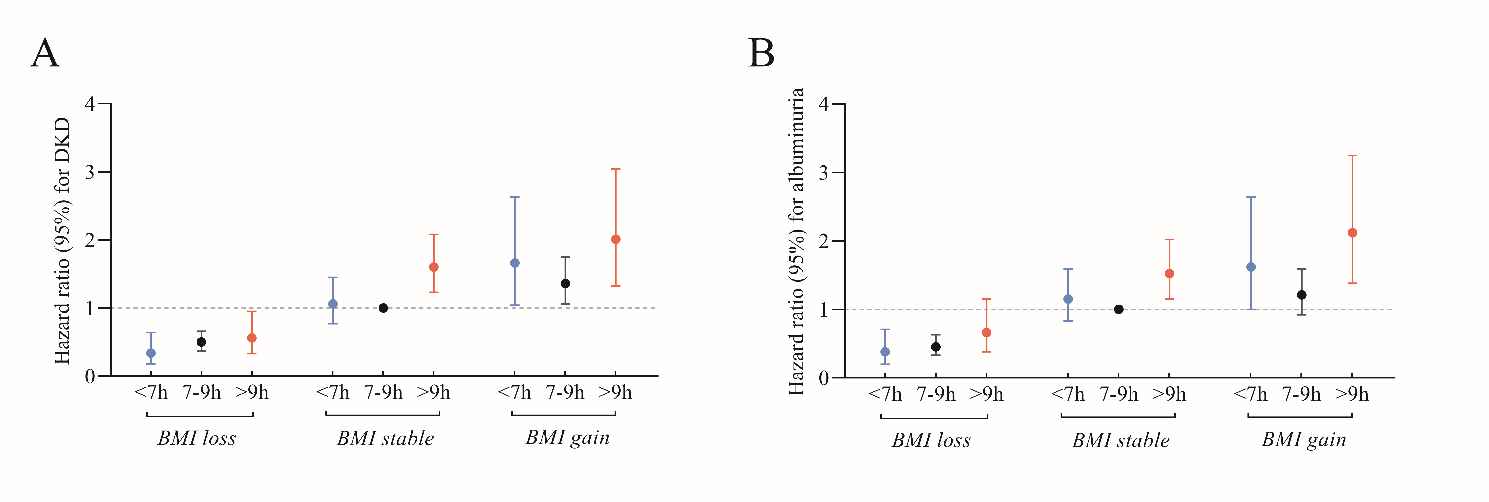
**Figure S5. Relationship between sleep duration and risk of DKD (A) and albuminuria (B) among participants with varying changes in BMI.

Individuals with a BMI reduction of 5.0% or higher were designated as the “BMI loss” group, the “BMI gain” group consisted of individuals who experienced a BMI increase of 3.0% or higher. The participants in the “BMI stable” group had a BMI decrease of < 5.0% or an increase of < 3.0%. Those who slept 7-9 hours/day and had stable BMI during follow-up were referenced. All models were adjusted for age, sex, duration of diabetes, HbA1c, smoking status, alcohol intake, eGFR, use of ACEI/ARB, history of hypertension, CVD and cancer, and sleep quality.

| **Table S1. Characteristics of BMI changes in the different groups according to changes in BMI or BMI status** | | | | | | |
| --- | --- | --- | --- | --- | --- | --- |
|  | N | Mean | SD | Median | Interquartile range | |
| Changes in BMI, (%) |  |  |  |  |  |  |
| Stable (middle thirds of changes in BMI) | 979 | -1.12 | 1.02 | -1.01 | -2.00 | 0.00 |
| Loss (lower thirds of changes in BMI) | 979 | -6.99 | 3.54 | -6.12 | -8.51 | -4.50 |
| Gain (upper thirds of changes in BMI) | 978 | 4.12 | 4.05 | 3.03 | 1.53 | 5.65 |
|  |  |  |  |  |  |  |
| Changes in BMI status, (%) |  |  |  |  |  |  |
| Remained normal | 938 | -0.82 | 4.83 | -0.85 | -3.61 | 1.74 |
| Remained overweight or obese | 1660 | -1.26 | 4.99 | -0.79 | -4.09 | 1.45 |
| Became normal | 231 | -7.78 | 4.45 | -7.26 | -10.04 | -4.57 |
| Became overweight or obese | 107 | 6.89 | 6.81 | 5.66 | 2.99 | 8.51 |
|  |  |  |  |  |  |  |
| Changes in BMI, (%) |  |  |  |  |  |  |
| Stable (changes in BMI between -5% and 5%) | 1806 | -0.96 | 2.07 | -0.75 | -2.58 | 0.43 |
| Loss (changes in BMI ≤ - 5%) | 636 | -8.58 | 3.45 | -7.56 | -9.81 | -6.23 |
| Gain (changes in BMI ≥ 3%) | 494 | 6.64 | 4.36 | 5.61 | 3.97 | 7.94 |

## Table S2. Risks of DKD and albuminuria according to sleep duration and BMI

|  | **Habitual sleep duration, h/day** | | |
| --- | --- | --- | --- |
|  | < 7 | 7 - 9 | > 9 |
| **DKD incidence** |  |  |  |
| Normal | 0.85 (0.54-1.33) | 1.00 (Ref) | 1.30 (0.88-1.92) |
| Overweight | 1.24 (0.84-1.84) | 1.16 (0.91-1.48) | 2.12 (1.52-2.94) |
| Obese | 1.57 (0.98-2.50) | 1.62 (1.22-2.16) | 1.83 (1.17-2.86) |
|  |  |  |  |
| **Albuminuria incidence** |  |  |  |
| Normal | 0.95 (0.58-1.54) | 1.00 (Ref) | 1.42 (0.93-2.15) |
| Overweight | 1.50 (1.00-2.26) | 1.24 (0.95-1.62) | 2.45 (1.72-3.48) |
| Obese | 1.81 (1.11-2.93) | 1.83 (1.34-2.49) | 2.06 (1.27-3.35) |

Note: Data are hazard ratios (95% CIs). BMI was divided into three categories: normal weight, < 24 kg/m^2^; overweight, 24-27.9 kg/m^2^ and obese, ≥ 28 kg/m^2^. Models were adjusted for age, sex, duration of diabetes, HbA1c, smoking status, alcohol intake, eGFR, use of ACEI/ARB, history of hypertension, CVD and cancer, and sleep quality.

## Table S3. Risks of DKD and albuminuria according to sleep duration and changes in BMI

|  | **Habitual sleep duration, h/day** | | |
| --- | --- | --- | --- |
|  | < 7 | 7 - 9 | > 9 |
| **DKD incidence** |  |  |  |
| Loss (lower thirds of changes in BMI) | 0.50 (0.31-0.82) | 0.61 (0.47-0.80) | 0.78 (0.51-1.20) |
| Stable (middle thirds of changes in BMI) | 0.96 (0.61-1.53) | 1.00 (Ref) | 1.53 (1.04-2.24) |
| Gain (upper thirds of changes in BMI) | 1.58 (1.08-2.29) | 1.37 (1.08-1.74) | 2.04 (1.48-2.83) |
|  |  |  |  |
| **Albuminuria incidence** |  |  |  |
| Loss (lower thirds of changes in BMI) | 0.55 (0.34-0.91) | 0.57 (0.43-0.77) | 0.91 (0.59-1.41) |
| Stable (middle thirds of changes in BMI) | 0.99 (0.61-1.60) | 1.00 (Ref) | 1.24 (0.80-1.90) |
| Gain (upper thirds of changes in BMI) | 1.60 (1.09-2.36) | 1.23 (0.95-1.59) | 2.09 (1.48-2.93) |

Note: Data are hazard ratios (95% CIs). Participants with the lowest change in BMI by one-third were classified as the “loss” group; those with the middle one-third change were classified as the “stable” group; and the upper thirds were classified as the “gain” group. Models were adjusted for age, sex, duration of diabetes, HbA1c, smoking status, alcohol intake, eGFR, use of ACEI/ARB, history of hypertension, CVD and cancer, and sleep quality.

## Table S4. Risks of DKD and albuminuria according to sleep duration and changes in BMI status

|  | **Habitual sleep duration, h/day** | | |
| --- | --- | --- | --- |
|  | < 7 | 7 - 9 | > 9 |
| DKD incidence |  |  |  |
| Remained normal | 0.88 (0.55-1.43) | 1.00 (Ref) | 1.28 (0.83-1.97) |
| Remained overweight or obese | 1.75 (1.24-2.46) | 1.57 (1.23-2.02) | 2.46 (1.79-3.39) |
| Became normal | 0.00 (0.00-Inf) ^*^ | 0.58 (0.34-0.97) | 1.14 (0.53-2.46) |
| Became overweight or obese | 1.19 (0.29-4.84) | 1.99 (1.20-3.33) | 2.49 (1.14-5.40) |
|  |  |  |  |
| Albuminuria incidence |  |  |  |
| Remained normal | 0.95 (0.57-1.58) | 1.00 (Ref) | 1.36 (0.86-2.16) |
| Remained overweight or obese | 1.98 (1.39-2.84) | 1.65 (1.26-2.16) | 2.68 (1.90-3.76) |
| Became normal | 0.00 (0.00-Inf) ^*^ | 0.58 (0.33-1.03) | 1.33 (0.58-3.07) |
| Became overweight or obese | 1.39 (0.34-5.68) | 1.57 (0.85-2.90) | 2.39 (1.03-5.52) |

Note: Data are hazard ratios (95% CIs). Participants with normal BMI at both baseline and the last examination were classified as the “Remained normal BMI” group, “Remained overweight or obese” for those who were overweight or obese at both examinations, “Became normal BMI” for those who were overweight or obese at baseline but had a normal BMI at the last examination, and “Became overweight or obese” for those with a normal BMI at baseline but were overweight or obese at the last examination.

Models were adjusted for age, sex, duration of diabetes, HbA1c, smoking status, alcohol intake, eGFR, use of ACEI/ARB, history of hypertension, CVD and cancer, and sleep quality.

^*^ The group with short sleep patterns that transitioned to a normal BMI did not have any participants develop DKD.

## Table S5. Risks of DKD and albuminuria according to sleep duration and changes in BMI

|  | Habitual sleep duration, h/day | | |
| --- | --- | --- | --- |
|  | < 7 | 7 - 9 | > 9 |
| DKD incidence |  |  |  |
| Loss (changes in BMI ≤ - 5%) | 0.34 (0.18-0.64) | 0.61 (0.47-0.80) | 0.78 (0.51-1.20) |
| Stable (changes in BMI between -5% and 3%) | 1.06 (0.77-1.45) | 1.00 (Ref) | 1.53 (1.04-2.24) |
| Gain (changes in BMI ≥ 3%) | 1.66 (1.04-2.63) | 1.37 (1.08-1.74) | 2.04 (1.48-2.83) |
|  |  |  |  |
| Albuminuria incidence |  |  |  |
| Loss (changes in BMI ≤ - 5%) | 0.38 (0.20-0.71) | 0.57 (0.43-0.77) | 0.91 (0.59-1.41) |
| Stable (changes in BMI between -5% and 3%) | 1.15 (0.83-1.59) | 1.00 (Ref) | 1.24 (0.80-1.90) |
| Gain (changes in BMI ≥ 3%) | 1.62 (1.00-2.64) | 1.23 (0.95-1.59) | 2.09 (1.48-2.93) |

Note: Data are hazard ratios (95% CIs). Individuals with a BMI reduction of 5.0% or higher were designated as the “loss” group; the “gain” group consisted of individuals who experienced a BMI increase of 3.0% or higher; the participants in the “stable” group had a BMI decrease of < 5.0% or an increase of < 3.0%. Models were adjusted for age, sex, duration of diabetes, HbA1c, smoking status, alcohol intake, eGFR, use of ACEI/ARB, history of hypertension, CVD and cancer, and sleep quality.

**
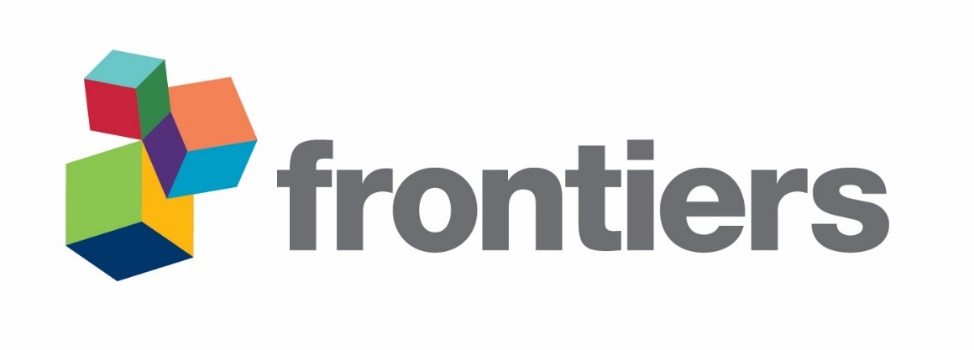
**
